# Predicting nucleosome positioning using a duration Hidden Markov Model

**DOI:** 10.1186/1471-2105-11-346

**Published:** 2010-06-24

**Authors:** Liqun Xi, Yvonne Fondufe-Mittendorf, Lei Xia, Jared Flatow, Jonathan Widom, Ji-Ping Wang

**Affiliations:** 1 Department of Statistics, Northwestern University, Evanston, IL 60208, USA; 2 Department of of Biochemistry, Molecular Biology and Cell Biology, Northwestern University, Evanston, IL 60208, USA; 3 Department of Electrical Engineering and Computer Science, Evanston, IL 60208, USA; 4 Clinical and Translational Sciences Institute, Northwestern University, Chicago, 60611, USA

## Abstract

**Background:**

The nucleosome is the fundamental packing unit of DNAs in eukaryotic cells. Its detailed positioning on the genome is closely related to chromosome functions. Increasing evidence has shown that genomic DNA sequence itself is highly predictive of nucleosome positioning genome-wide. Therefore a fast software tool for predicting nucleosome positioning can help understanding how a genome's nucleosome organization may facilitate genome function.

**Results:**

We present a duration Hidden Markov model for nucleosome positioning prediction by explicitly modeling the linker DNA length. The nucleosome and linker models trained from yeast data are re-scaled when making predictions for other species to adjust for differences in base composition. A software tool named NuPoP is developed in three formats for free download.

**Conclusions:**

Simulation studies show that modeling the linker length distribution and utilizing a base composition re-scaling method both improve the prediction of nucleosome positioning regarding sensitivity and false discovery rate. NuPoP provides a user-friendly software tool for predicting the nucleosome occupancy and the most probable nucleosome positioning map for genomic sequences of any size. When compared with two existing methods, NuPoP shows improved performance in sensitivity.

## Background

Most eukaryotic genomic DNA is wrapped in nucleosomes, which occlude and strongly distort the wrapped DNA. Accumulating evidence shows that the DNA sequence itself is highly predictive of nucleosome positioning in vivo [1-7], and that nucleosome positioning is closely related to chromosome functions [1,8-11]. A fast software tool for predicting nucleosome positioning is highly desirable.

Several statistical methods for nucleosome positioning prediction have been proposed in the literature. In [2] a method was proposed based on cross-correlation with a nucleosome DNA sequence signature. In [1] a Markov model was used together with consideration of steric exclusion and thermodynamic equilibrium. In [3], a support vector machine (SVM) was trained based on the differential *k*-mer usage between nucleosome and linker DNAs. In [4], the authors proposed an *N*-score model to discriminate nucleosome and linker DNAs using wavelet energies as covariates in a logistic regression model. In [5], a web-interface called "nuScore" for estimation of the affinity of histone core to DNA and prediction of nucleosome positioning was developed based on the DNA deformation energy score. In [6,7], the model from [1] was improved by incorporation of differential *k*-mer usage (most notably, poly(dA:dT) tracts, which are strongly disfavored by nucleosomes). This model can be further improved by accounting for nucleosome-nucleosome interaction [12].

While the nucleosomal features are universal, eukaryotic genomes vary in nucleosomal repeat length [13] and base composition. The nucleosomal repeat length is dictated by the length distribution of linker DNAs that separate neighboring nucleosomes, and it determines the overall nucleosome density in the chromatin fiber. The contribution of this paper is a duration Hidden Markov Model and a software tool called NuPoP for genome-wide nucleosome positioning prediction. We show that incorporation of linker length information can achieve better sensitivity in prediction. In addition, we propose a re-scaling method to adjust for base composition variation when using yeast models to make predictions for other species. A relatively superior performance of this approach is established by comparing it with other existing tools.

## Methods

### Model

The hidden Markov model (HMM) has been known for decades. An excellent and famous tutorial is Rabiner's 1989 paper [14], in which the model, algorithms, and applications were carefully and thoroughly reviewed. A conventional HMM implicitly assumes a geometric duration distribution for each state, which can be wrong in real applications. Modeling the explicit duration of each state can improve the prediction of HMMs (e.g., [14-17]). We model each chromosomal DNA sequence with a duration hidden Markov model (dHMM) of two oscillating states: nucleosome (*N*) and linker (*L*), where the nucleosome state has a fixed length of 147 bp, and the linker state has a variable length. We assume that at the end of each state, the chain must transit to the other state; additionally, a complete chromatin sequence must start with and end in a linker state. We trained a 4th order time-dependent Markov chain for the the *N *state, and a homogeneous 4th order Markov chain for the *L *state to distinguish the *k*-mer usage preferences for *k *up to 5 between the nucleosome and linker states as shown in other methods, e.g., [3,6] (see below for details).

Let **e **= *e*_1_, ..., *e*_147 _be a nucleosome DNA sequence. Let *P*_*N *_be the probability of observing **e **as a nucleosome, computed as the product of probabilities for both Watson and Crick strands under the 4th order Markov Chain model. We assume that the linker DNA length of a given species has an unknown distribution *F*_*L*_(*k*) defined for *k *= 1, ..., τ_*L *_(the maximum linker length we allow). An observed linker DNA sequence **e **= *e*_1_, ..., *e*_*k *_carries two pieces of information, the length is *k *bp, and given which, the emitted letters are *e*_1_, ..., *e*_*k*_. Let *G*_*L*_(**e**|*k*) denote the homogeneous Markov chain model for the linker DNA (again including both strands). Then observing **e **as a linker DNA has probability

Suppose **x **= *x*_1_, ..., *x*_*n *_is a genomic DNA sequence of length *n*, where *x*_*i *_= *A*/*C*/*G*/*T*. Let **z **= *z*_1_, ..., *z*_*n *_be the corresponding hidden state path, where *z*_*i *_= 1 if *x*_*i *_is covered by a nucleosome state, and 0 otherwise. Suppose that the path **z **= *z*_1_, ..., *z*_*n *_partitions **x **into *k *consecutive nucleosome or linker state blocks, in which the nucleosome blocks have a uniform length of 147 bp, whereas the length of linker blocks may vary. We denote these blocks as **y **= **y+**, ..., **y_B_**, and their state identification as **s **= *s*_1_, ..., *s*_*B*_, where *s*_*i *_= 1 if **y**_*i *_is nucleosome state, and 0 otherwise. The probability of observing (**x**, **z**) is given by

where π_0_(*s*_1_) and π*e*(*s*_*B*_) stand for the probabilities that the chain initializes and ends with state *s*_1 _and *s*_*k *_respectively, and *I *is an indicator function. Since we assume that a complete chromatin sequence must start with and end in a linker state, π_0_(*s*_1 _= 0) = π_*e*_(*s*_*B *_= 0) = 1. We define the nucleosome occupancy at a specific position *i*, denoted *o*_*i*_, as the posterior probability that *z*_*i *_= 1, i.e.,

We also define the histone binding affinity score at position *i *as the log likelihood ratio for the region *x*_*i*_-73, ... *x*_*i*_, ..., *x*_*i*_+73 to be a nucleosome vs. a linker, i.e.,

Given the models *P*_*N*_, *G*_*L *_and *F*_*L*_, the optimal path **z **can be found by the standard Viterbi algorithm, and the nucleosome occupancy score can be estimated using forward and backward algorithms.

### Data and model training

We utilized the 503,264 yeast nucleosome DNA reads from 454 pyrosequencing published in [6] for model training and assessment. Among 371,914 reads that each were mapped to a unique region of the yeast genome, we first selected reads of length between 146 and 149 bp. If multiple such reads existed for the same nucleosome, we selected the one with the highest BLAST score. The resulting non-redundant set of 18,547 nucleosome sequences were center aligned to train the nucleosome model *P*_*N*_. The 4th order time dependent Markov chain can be defined by the base composition at the first position *qN*(*x*_1_), and the transitional probabilities *qN*(*x*_2_|*x*_1_), *qN*(*x*_3_|*x*_1_, *x*_2_), *qN*(*x*_4_|*x*_1_, *x*_2_, *x*_3_), *qN*(*x*_*k*_|*x*_*k*-4_, *x*_*k*_-3, *x*_*k*-2_, *x*_*k*-1_), for *k *= 5, ..., 147, *x*_*i *_= A/C/G/T, *i *= 1, ..., 147, where the subscript *k*, *i *index the positions within a nucleosome. These probabilities are trained using the corresponding observed fractions or conditional fractions based on the center alignment, with a three bp moving average (as explained in [1,18]). We further identified 8,090 reads-free regions of length 7-500 bp to train the linker state model *G*_*L*_. The 4th order homogeneous Markov model for the linker DNAs can be completely defined by the stationary base composition *qL*(*xi*), and the transition probabilities *qL*(*x*_*i*_|*x*_*i*-1_), *qL*(*x*_*i*_|*x*_*i*-1_, *x*_*i*-2_), *qL*(*x*_*i*_|*x*_*i*-1_, *x*_*i*-2_, *x*_*i*-3_), *qL*(*x*_*i*_|*x*_*i*-1_, *x*_*i*-2_, *x*_*i*-3_, *x*_*i*-4_). By "homogeneous", we mean that these probabilities are all constants as functions of *i*. These probabilities were trained using their observed values as in the nucleosome model. For example, *qL*(*x*_*i*_|*x*_*i*-1_, *x*_*i*-2_, *x*_*i*-3_, *x*_*i*-4_) was trained by calculating the fraction of occurrences of transitions from any four letters to the fifth letter in the selected putative linker DNA sequences.

Our initial nucleosome/linker model was trained using the yeast data. A complication arises when predicting nucleosomes for other species because organisms may differ significantly in their DNA base composition. We propose to scale up or down the probabilities in the Markov models by a factor determined by the difference of the base composition between the current species and yeast. For example, in *C. elegans*, the fraction of A plus T bases is 0.645 compared to 0.617 in yeast. For a specific transition probability *qN*(*A*|....) at any specific nucleosomal position defined for yeast, we scaled it up as *qN*(*A*|....) × 0.645/0.617 for the corresponding transition probability at that given position for *C. elegans*. Likewise the transition probabilities for G/C will be scaled down by a factor of 0.355/0.383.

All the re-scaled probabilities are then normalized. The same re-scaling applies to the linker model. We shall use simulations below to show that re-scaling improves prediction regarding sensitivity and false discovery rate. Using the trained nucleosome model (*P*_*N*_) and linker model (*G*_*L*_), we further train the linker DNA length distribution as follows. We assume that the linker DNAs in any given species or cell type have a maximum length τ_*L *_= 500 bp.

This algorithm contains the following steps:

1. Initialize the algorithm with a uniform distribution for *F*_*L*_(*k*) for *k *= 1, ... τ_*L *_where τ_*L *_is the maximum allowable linker length.

2. Use the forward and backward algorithm to obtain the posterior expectation of *F*_*L*_(*k*) for each *k*. For a sequence **x **= *x*_1_, ..., *x*_*n*_, let *n*_*k *_be the number of linker DNAs of length *k*. Then

for *k *= 1, ..., τ_*L *_.(1)

for *k *= 1, ..., τ_*L *_.

3. Update the empirical linker length distribution from step 2 using a kernel smoothing method as follows:(2)

where *K *is the standard Gaussian kernel and *h *is the bandwidth parameter optimally chosen by the leave-one cross-validation method as in [19].

4. Use the updated linker length distribution from step 3 to compute the nucleosome occupancy and optimal positioning.

Compared to Viterbi training (i.e., using linker length predicted from the Viterbi algorithm), using the posterior expectation obtained in Eq. (1) combined with the kernel method in Eq. (2) performs overwhelmingly better in minimizing the summed square errors  (unpublished work [17]). In the developed software tool NuPoP, we have trained the linker DNA length distributions for 11 different species including human, mouse, rat, zebrafish, *D. melanogaster, C. elegans, S. cerevisiae, C. albicans, S. pombe; A. thaliana *and maize. The linker DNA length distribution (F_*L*_) for each species has been trained by scanning the corresponding genome sequences based on τ _*L *_= 500. We found that the re-scaled nucleosome and linker profiles, together with the trained linker length distribution, not only roughly recover the genome-wide base compositions, but also the dinucleotide frequencies for different species. The frequency of each single or di-nucleotide in simulated genomes typically differs by ≤ 1% from that observed in the corresponding real genomes (results not shown). As different cell types from the same organism (with the same genome) can exhibit quite different linker DNA length distributions [13], a useful future refinement would utilize high quality nucleosome maps for the given cell type, when such data become available.

### Software tools

We have developed a software tool called NuPoP, implemented in three different formats: an R package tested for Windows XP, Linux and Mac OS X; a stand-alone Fortran program; and an NuPoP web server, all available from http://nucleosome.stats.northwestern.edu. The R package is built upon the Fortran program. It provides additional handy functions to visualize the resulting Viterbi (most probable nucleosome position map) and nucleosome occupancy predictions. Both the R package and Fortran program can handle a genomic sequence of any length with a RAM demand <400 M bytes. The predicted results are stored locally in the working directory. The web server provides an interface through which the user can submit their own sequence up to 500 K bp in length for fast online prediction. When making a prediction, the user is required to specify which species the genomic sequence is from. If the species is not on the list, NuPoP will calculate the base composition of the input DNA sequence and then choose the nucleosome and linker models from a species that has the most similar base composition. An alternative model with a 1st order time-dependent Markov chain for the nucleosome state and a homogeneous 1st order Markov model for the linker state, trained in the same way, is also implemented in NuPoP as an option.

## Results

### Updating *F*_*L *_improves prediction

Updating the linker length distribution not only helps recover the true nucleosome density, but also improves prediction. We demonstrated this by simulation as follows. We simulated 10 genomic sequences with the 4th and 1st order yeast models respectively, each containing 10,000 nucleosomes and 10,001 linkers. The linker DNA length was simulated from a Normal distribution (*μ *= 100, *σ *= 20) and a Gamma distribution (α = 1, β = 1/40). If a nucleosome is predicted within ±35 bp of a true nucleosome, we define it as a correct positive prediction. The rate of the correct positive prediction, referred to as *sensitivity*, is defined as the percentage of the 10,000 nucleosomes that are correctly predicted. In addition, we include the false discovery rate (FDR), defined as the fraction of the predicted nucleosomes that reside > ±35 bp away from any true nucleosomes, as the second measure for model performance. In analogy to statistical hypothesis testing, the sensitivity measures the power of prediction, while the FDR measures the fraction of type I errors in the positive claims. The results are presented in Table 1 and 2. In both cases, the linker length was initialized as a uniform distribution with τ_*L *_= 200. Compared to the Gamma distribution, the Normal distribution is relatively flatter. Thereby updating the linker length distribution did not significantly change the total number of predicted nucleosomes (or nucleosome density). The sensitivity increased on average by ~ 4-5% and the FDR dropped by ~ 5% after one update (for both the first and fourth order models). Further updating continued to improve the prediction until it stabilized after four iterations. In contrast, the Gamma model is much more skewed. The uniform linker length distribution resulted in an under-estimation of the total number of nucleosomes. By four updatings, the sensitivity increased by 8%, while FDR remained relatively more stable.

**Table 1:** Updating linker length improves prediction - Normal linker length model

		1st order				4th order	
update	total	sensitivity(%)	FDR(%)	update		sensitivity (%)	FDR(%)
0	10215 (14)	72 (0.5)	30 (0.5)	0	10253 (15)	75 (0.4)	27 (0.4)
1	10210 (12)	76 (0.5)	25 (0.5)	1	10231 (18)	80 (0.5)	22 (0.5)
4	10120 (19)	83 (0.7)	18 (0.8)	4	10131 (16)	85 (0.6)	16 (0.7)

Total predictions, sensitivity, and false discovery rate (FDR) are the averages (standard deviations in parentheses) based on 10 repeated simulations. For each simulation a genomic sequence consisting of 10000 nucleosomes and 10001 linkers were simulated using the 1st and 4th order yeast models. The linker length distribution was initialized as uniform on 1,..., 200, and was iteratively updated in the dHMM. Results are shown after 0, 1, and 4 updates.

**Table 2:** Updating linker length improves prediction - Gamma linker length model

		1st order				4th order	
update	total	sensitivity(%)	FDR(%)	update	total	sensitivity (%)	FDR(%)
0	8670 (22)	59 (0.7)	32 (0.7)	0	8896 (27)	64 (0.5)	28 (0.5)
1	9347 (25)	65 (0.8)	30 (0.7)	1	9550 (42)	70 (0.6)	26 (0.6)
4	9833 (39)	67 (0.7)	31 (0.7)	4	9880 (35)	72 (0.5)	27 (0.6)

Total predictions, sensitivity, and false discovery rate (FDR) are the averages (standard deviations in parentheses) based on 10 repeated simulations. For each simulation a genomic sequence consisting of 10000 nucleosomes and 10001 linkers were simulated using the 1st and 4th order yeast models. The linker length distribution was initialized as uniform on 1,..., 200, and was iteratively updated in the dHMM. Results are shown after 0, 1, and 4 updates.

We also observe that under the same setting and condition, the fourth order model performs slightly but uniformly better than the first order model in both sensitivity and FDR. This is given that the true models are known and we scan the sequence using the true models. In theory, the first order Markov chain model is nested in the fourth order model. Therefore if the true model is the first order, a well trained fourth order model will have the same prediction power as the first order model, but not vice versa. Since training a higher order Markov chain model requires more data, inadequate training can undermine the prediction power.

### Re-scaling vs. not Re-scaling

To illustrate the advantages of re-scaling, we re-scaled the yeast profiles according to the base composition of the maize genome (G/C scaling factor in maize is 1.2). Using the re-scaled profiles we simulated 10 genomic sequences that each contain 10,000 nucleosomes and 10,001 linkers. The linker DNA length followed the same two distributions as in Table 1 and 2. We compare the prediction results from the scaled and non-scaled yeast profiles in Table 3 and 4. We found using the re-scaled ("correct") profile yields a lower FDR than using the yeast profile. In addition, updating the linker length under the correct profile consistently improves the sensitivity and FDR until prediction stabilizes. In contrast, while using the yeast profile to scan the simulated maize-like genome, the prediction drastically deteriorates as the linker length updating proceeds. The same simulation was repeated on other species including human and *C. elegans*, where the base composition is similar to yeast (A/T scaling factor is 0.96 for *C. elegans*, and 1.03 for human). Unsurprisingly, the results from the scaled yeast profile were still better than those from the original yeast profile in terms of both sensitivity and FDR, while the difference is much smaller than for the maize case (results not shown).

**Table 3:** Re-scaling models improves prediction - Normal linker length model

model	update	re-scaled total	sensitivity (%)	FDR(%)	update	total	sensitivity (%)	FDR(%)
1st	0	10266 (12)	71 (0.4)	31 (0.4)	0	13272 (24)	59 (0.5)	55 (0.4)
	1	10279 (15)	76 (0.4)	27 (0.4)	1	14803 (25)	53 (0.4)	64 (0.3)
	2	10240 (19)	79 (0.3)	23 (0.3)	2	15383 (23)	51 (0.4)	67 (0.3)
4th	0	10280 (16)	74 (0.3)	28 (0.3)	0	12785 (28)	63 (0.4)	51 (0.4)
	1	10267 (20)	79 (0.4)	24 (0.5)	1	14065 (25)	58 (0.3)	59 (0.3)
	2	10220 (24)	81 (0.4)	20 (0.5)	2	14591 (24)	55 (0.4)	62 (0.3)

Total predictions, sensitivity, and false discovery rate (FDR) are the averages (standard deviations in parentheses) based on 10 repeated simulations. For each simulation a maize-like genomic sequence consisting of 10000 nucleosomes and 10001 linkers were simulated using the re-scaled 1st and 4th order yeast models. Each sequence was scanned using the true models (re-scaled, 1st or 4th order) and the yeast models with an initial uniform linker length distribution on 1,..., 200. The results after 0, 1, 2 updates of linker length distribution are compared.

**Table 4:** Re-scaling models improves prediction - Gamma linker length mode l

model	update	re-scaled total	sensitivity (%)	FDR(%)	update	total	sensitivity (%)	FDR(%)
1st	0	8746 (28)	60 (0.7)	31 (0.6)	0	10640 (19)	70 (0.3)	35 (0.3)
	1	9471 (42)	67 (0.7)	30 (0.6)	1	11513 (14)	60 (0.6)	48 (0.6)
	2	9787 (38)	68 (0.5)	30 (0.4)	2	11812 (18)	55 (0.4)	53 (0.3)
4th	0	8886 (18)	63 (0.3)	29 (0.4)	0	10461 (25)	73 (0.7)	30 (0.7)
	1	9533 (26)	70 (0.8)	27 (0.8)	1	11190 (32)	66 (0.7)	41 (0.7)
	2	9775 (33)	72 (0.8)	27 (0.9)	2	11443 (26)	63 (0.5)	45 (0.5)

Total predictions, sensitivity, and false discovery rate (FDR) are the averages (standard deviations in parentheses) based on 10 repeated simulations. For each simulation a maize-like genomic sequence consisting of 10000 nucleosomes and 10001 linkers were simulated using the re-scaled 1st and 4th order yeast models. Each sequence was scanned using the true models (re-scaled, 1st or 4th order) and the yeast models with an initial uniform linker length distribution on 1,..., 200. The results after 0, 1, 2 updates of linker length distribution are compared.

### NuPoP vs. other software tools

**Figure 1:**
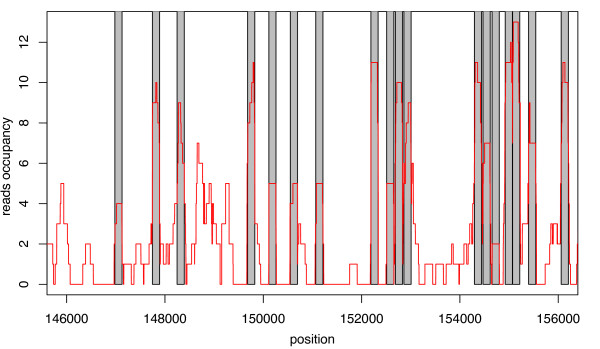
**A plot of the experimentally defined reads occupancy score (red curve) for a region of yeast chromosome 4 showing the selected well-defined nucleosomes (grey shaded bars)**.

**Figure 2:**
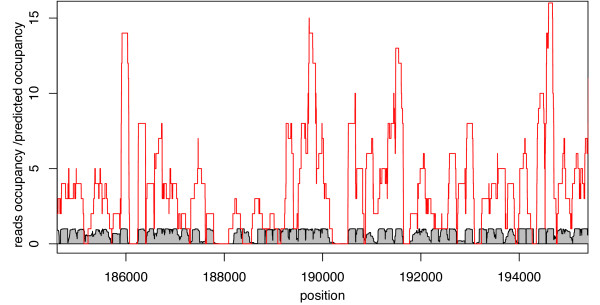
**A snapshot of predicted nucleosome occupancy from NuPoP (shaded grey) compared with the experimentally obtained reads-occupancy (red)**.

We briefly assess the prediction performance of NuPoP by comparing it with two existing methods: the N-score method of [4] (results kindly provided by Dr. G. Yuan, personal communication) and the Markov model/thermodynamic equilibrium method of [7] (to be called MM/TE method below). As the exact genome-wide nucleosome positioning map is unknown, we utilize the 371,914 454 high-throughput sequence reads to identify well-defined nucleosomes. We first selected sequences of length between 130-160 bp and constructed a reads-based occupancy map. The reads-occupancy score at a specific position is defined as the number of reads that covered this position. Then we calculated the moving average of this reads occupancy score using a 147 bp window. A sharp peak in the average occupancy curve indicates a nucleosome with well-defined positioning. Considering that the average linker DNA length is 20 bp in yeast [13], we quantified the sharpness of the peak by calculating the slope from the peak point to the up-/down-stream 20 bp point on the average occupancy curve. We set one condition for the peak to be selected as the center of a nucleosome to be that the absolute value of slope from either side should be > 0.01. Secondly, we required that the peak height itself must be ≥ 1.9, i.e., a well-defined nucleosome must be testified by at least two well-overlapped reads. We chose the threshold value as 1.9 instead of 2.0 because the overlap of the two reads can be less than 147 bp, resulting in a peak on the moving average curve slightly lower than 2.0. With these criteria, a total of 20,471 well-defined nucleosomes are selected from the 16 chromosomes of yeast. A snapshot of a region with many selected well-defined nucleosomes is presented in Figure 1. Figure 2 provides a snapshot of nucleosome occupancy predicted by NuPoP together with the reads-occupancy.

**Figure 3:**
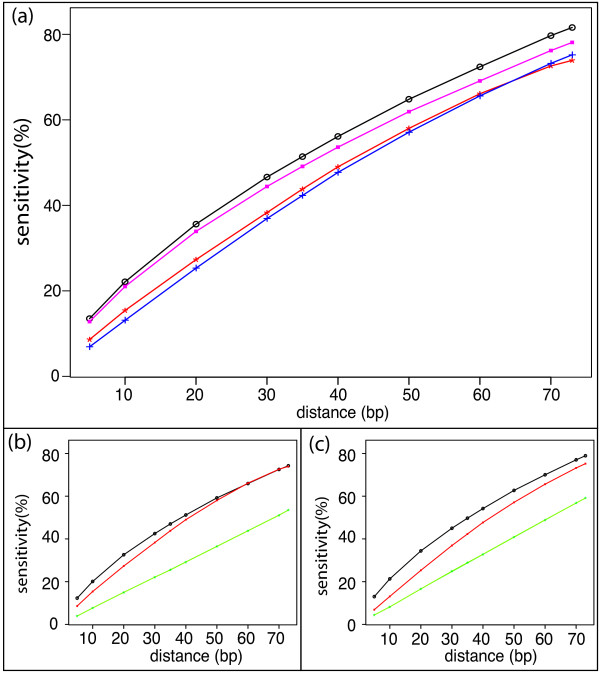
**Comparing sensitivity of NuPoP predictions with existing methods**. The sensitivity is assessed based on 20,471 well-defined nucleosomes from 454 nucleosomes reads. We call a prediction correct if a nucleosome is predicted within +/- k bp distance (X-axis) of a well-defined nucleosome (center to center) for k = 5 to 73. (a) Sensitivity plot of NuPoP (black) compared with N-score method (blue), MM/TE method (red), and the duration Hidden Markov Model using a uniform distribution on 1,...,500 (pink). The random expectation curve is not calculated because the total number of predictions varies in different methods. (b) Sensitivity plot of NuPoP (black) compared with MM/TE method (red) and random expectation (green) while controlling the total predictions to be the same as MM/TE method. Green: random expectation. (c) Sensitivity plot of NuPoP (black) compared with N-score method (red) and random expectation (green) while controlling the total predictions to be the same as N-score method.

We first assess the sensitivity of predictions from NuPoP using the well-defined control set. If there is a predicted nucleosome within ±*k *bp of any well-defined nucleosomes (center to center), we count this as one correct prediction. We varied *k *from 5, 10, ... 70, 73 to investigate the sensitivity behavior at different precision thresholds. The N-score model predicted 48,394 nucleosomes. The current software tool for MM/TE method does not provide Viterbi predictions, but only the nucleosome occupancy scores. Therefore we calculated the moving average of the occupancy score using a 147 bp moving window. The resulting peaks were treated as the centers of predicted nucleosomes. If two peaks reside within 127 bp, we discarded the one with smaller moving average of occupancy score. This procedure identified 43,979 predicted nucleosomes (if we had required two nucleosomes to be 147 bp away, even fewer nucleosomes would have been identified). Likewise, using the occupancy scores from NuPoP, we identified 52,327 and 51,380 predicted nucleosomes under the 4th and 1st order models respectively. In Figure 3a, we compare the sensitivity estimates from the 4th order model of NuPoP with the N-score and MM/TE methods at different threshold values of prediction accuracy (the fourth order model from NuPoP performs better, but very slightly, than the first order model. Hence the latter was omitted in Figure 3 for better presentation). As the sensitivity tends to increase with an increase in the total predictions, we further selected 43,979 and 48,394 best predictions from NuPoP model (here "best" is in the sense of the largest sum of occupancies over 147 bp) to compare with the N-score method and in Figure 3b and 3c respectively.

The sensitivity results suggest that the predictions from NuPoP outperforms the other two methods in two senses. Firstly, the sensitivity from NuPoP is 4.9-8.3% higher than the other two methods at different threshold values (Figure 3a). Secondly, while controlling the total predictions to be the same, NuPoP has ~ 3.2-5.3% higher sensitivity than the MM/TE method (Figure 3b), when the precision threshold is ≤ ±35. As the precision threshold gets less stringent, the difference attenuates and eventually vanishes. The contrast between NuPoP and the N-score method is even larger as shown in Figure 3c.

As a further comparison, we computed the predictions of the dHMM method under a uniform linker length distribution defined on 1, 2,..., 500. This method predicted 48,334 nucleosomes under the 4th order models, achieving a sensitivity 3.0-6.6% higher than the MM/TE method (Figure 3a). When controlling the total predictions to be the same as MM/TE method or N-score method, the resulting sensitivity curve almost perfectly overlaps with that from NuPoP. Therefore we omitted these results from Figure 3b and 3c.

One could further attempt to evaluate the false positive rate (FPR), measuring the fraction of linker regions that were falsely classified as nucleosome regions (or similarly the false discovery rate, FDR). This task requires well-defined linker regions. A problem, however, is that the average length of linker DNAs in yeast (20 bp; [13]) is smaller than the dispersion in lengths of the nucleosome DNAs as isolated biochemically (which is often 30-50 bp full width at half maximum, notwithstanding that the nucleosome as defined crystallographically has precisely 147 bp of DNA). Thus existing nucleosome maps lack the precision needed to define such short linker DNAs. Moreover, various sampling biases such as the DNA sequence preferences of the micrococcal nuclease used to liberate nucleosomes biochemically (which preferentially cleaves A/T rich regions) could yield longer genomic regions that are free of recovered nucleosome DNA reads even if they are actually nucleosome occupied [20]. Attempts to evaluate the FPR given these problems in the data could result in misleading conclusions. For these reasons, FPR evaluation is not pursued in this paper.

## Discussion

The duration Hidden Markov model proposed in this paper is a generic model for the oscillating structure of nucleosome and linker DNAs in chromatin fiber. The Markov models can be replaced by any other models for the nucleosome and linker states. The kernel method for linker length training is nonparametric and typically robust. We showed in the simulation that updating the linker length distribution iteratively improves sensitivity and FDR in prediction if appropriate nucleosome and linker models are used. In particular, the first iteration often achieves the most pronounced improvement. In contrast inappropriate nucleosome and linker models could lead to the opposite outcome, as shown in the simulation studies (Table 3 and Table 4). In reality, the genomic DNAs are complicated by their biological functions. The models trained based on typical nucleosomes or linkers may not well fit some special genomic regions like repeated elements. To avoid possible risks due to such complications, we trained the linker length distribution less greedily by using only one iteration in NuPoP.

Limitations may still exist in the model training and assessment used in this study. The MNase is known to have strong preference to cleave dinucleotides containing only A/T [21]. Consequently the MNase-mapped nucleosome sequences can be systematically biased in some regions. This bias could undermine the prediction power because of the dampened signal in the trained nucleosome model. The systematic bias may also exist in the well-defined nucleosomes, causing inaccuracy in sensitivity estimation. A better map of nucleosomes is highly desirable for both purposes. For species other than yeast, we currently lack high-quality genome-wide nucleosome sequence data (e.g., like the 454 reads) for model training and model validation. The advantages of the re-scaling method shown using simulation in this paper need to be further assessed once such high-quality data becomes available. Moreover, the results from different methods in this paper were all based on the default settings. The N-score method was originally trained based on a much smaller set of nucleosome and linker sequences. A better training using a larger set could improve this method's predictions. In addition, different settings in the N-score or MM/TE methods can lead to different predictions, which we did not further investigate here. Finally, the software for MM/TE method only provides the occupancy score. Different ways to call a predicted nucleosome based on the occupancy score might lead to different conclusions.

Finally, we address the question of which subset of the available 454 reads data might best be used for training the nucleosome model. In NuPoP, we trained the nucleosome model using selected non-redundant nucleosome reads of length within a short range (146-149 bp), to retain strong high resolution nucleosome sequence signatures, e.g., the _10 bp-periodic dinucleotide signals. As comparisons, we trained two additional nucleosome models: one using the selected non-redundant reads of length 122-177 bp (retaining the non-redundancy but yielding far more training data), and the other using all reads of length 122-177 bp. The resulting models both contain the k-mer usage information that distinguishes nucleosomes from linkers (e.g., [3,4]), while the dinucleotide signals in these models are much weaker due to poor alignment of these reads. Furthermore, as the reads count at a nucleosome site is heavily biased by the G/C content due to MNase specificity and other effects in the experiment, the model trained from the redundant reads tends to be over-enriched in G/C. When combined with the linker model from NuPoP, the two alternative nucleosome models yielded comparable sensitivity as NuPoP in predicting the approximate positioning of nucleosomes, assessed based on the 20,471 well-defined nucleosomes used above. This comparison, however, is not sensitive to spatial precision of the predictions. Therefore, we asked further, given that a nucleosome is predicted within ±73 of a true nucleosome, which model predicts the location more accurately? To investigate this, we simulated genomic sequences using the nucleosome and linker models from NuPoP. We compared the prediction from the three models and found that the true model with strong signals achieves much better prediction accuracy than the two alternative models. For example, 16.1% of the predictions from the true model were prefect (with 0 bp offset), compared to 8.7% and 5.9% respectively from the other two models (results not shown).

## Conclusions

The dHMM model proposed in this paper is effective in characterizing the oscillating structure of nucleosome and linker DNAs in chromatin fiber. Explicit modeling of linker length improves the prediction of nucleosome positioning regarding sensitivity. The developed software tool NuPoP provides a user-friendly interface for predicting nucleosome occupancy and the most probable nucleosomes positioning map genome-wide.

## Availability and requirements

NuPoP software tools are freely available from http://nucleosome.stats.northwestern.edu. The R package shall be made available through bioconductor http://www.bioconductor.org upon publication. To run the NuPoP Fortran stand-alone program, a Fortran compiler is required. For the NuPoP R package, an R version later than 2.9 is required.

## Authors' contributions

LXi did all the data analyses. JPW, JW, and LXi wrote the paper. JPW and JW directed the research. YFM conducted all lab work for data generation and validation. JPW and LXi developed the NuPoP software tools. LXia and JF implemented the NuPoP web-server. All authors read and approved the final manuscript.
